# Revealing a Novel Potential Pest of Plum Trees in the Caucasus: A Species Resembling the European Leaf-Mining *Stigmella plagicolella*, Nepticulidae #

**DOI:** 10.3390/insects15030198

**Published:** 2024-03-15

**Authors:** Jonas R. Stonis, Arūnas Diškus, Svetlana Orlovskytė, Viktorija Dobrynina

**Affiliations:** 1 State Research Institute Nature Research Centre, Akademijos g. 2, 08412 Vilnius, Lithuania; s.orlovskyte@gmail.com (S.O.); viktorija.dobrynina@gamtc.lt (V.D.); 2 Education Academy, Vytautas Magnus University, K. Donelaičio g. 58, 44248 Kaunas, Lithuania; diskus.biotaxonomy@gmail.com

**Keywords:** Insecta, leaf mines, Lepidoptera, mitotype network, Nepticulidae, new species, pests, potential pests, plum tree, *Stigmella*, taxonomy

## Abstract

**Simple Summary:**

While feeding, the larvae of pygmy moths (Nepticulidae) consume part of a leaf’s photosynthetic tissue, thus causing damage to the host plant. Although these insects play a natural role in terrestrial ecosystems, they are perceived as pests in horticulture. This study investigates the identity of a potential Nepticulidae pest infesting plum trees in the Caucasus, initially presumed to be the widespread European *Stigmella plagicolella* (Stainton). Contrary to expectations, our detailed taxonomic analysis revealed a new species of pygmy moth with distinct male genitalia morphology and molecular differences from its European counterpart. This discovery challenges previous records and highlights the need for accurate pest identification. We hypothesize that the proximity of the Caucasus to Europe emphasizes the importance of monitoring and preventing unintentional pest spread, particularly given potential climate change impacts on the distribution and behavior of pests. Our findings contribute to both practical pest management and scientific understanding of evolutionary processes shaping the Caucasian and European biota.

**Abstract:**

In instances of severe infestations, Nepticulidae larvae can inflict damage on cultivated plants. Previously, it was assumed that the *Prunus*-feeding Nepticulidae have continuous distribution from Europe to the neighboring Caucasus. During recent fieldwork in the Caucasus, leaf mines were found on plum trees that initially resembled those of *Stigmella plagicolella* (Stainton) in Europe. However, upon rearing the adults, significant differences emerged, leading to the hypothesis that a different *Prunus*-feeding species exists in the Caucasus; this challenges previous records in Western Asia. This paper presents the outcomes of our morphological, molecular, and statistical investigations, unveiling *S. colchica* sp. nov., a previously unknown potential plum-tree pest. Distinguished by male genitalia characteristics, the new species differs from *S. plagicolella*. The inter- and intraspecific divergences between *S. colchica* sp. nov. and *S. plagicolella* range from 3.5% to 6.02%. Moreover, the utilized delimitation algorithms reliably clustered two species separately, as does our mitotype network. A statistical analysis also shows a discernible trend between the leaf mines of *S. colchica* sp. nov. and *S. plagicolella*. This unexpected discovery not only documents a new potential pest, enhancing our understanding of the Caucasian fauna, but also contributes to the broader biological inventory.

## 1. Introduction

Revealing species diversity and identifying potential pests are crucial tasks, not only from a practical standpoint but also for gaining a better understanding of global biodiversity, the evolutionary history of the biosphere, and finding nature-inspired solutions for a sustainable future [1,2].

Nepticulidae (pygmy moths) is a morphologically, ecologically, and genetically distinctive cosmopolitan family of Lepidoptera [3,4,5,6]. Recently, the dynamics of species descriptions of the global fauna of pygmy moths were analyzed [7]. Over the last decade, the number of described Nepticulidae species increased from around 860 species [8] to 1014 species just before our study [6,9]. Valuable information on species diversity, the distribution, ecology, and morphology of Nepticulidae can be found in various monographs, books, and articles, particularly those cited in current publications [3,6,10,11,12,13,14].

The larvae of Nepticulidae are specialized plant miners, primarily targeting leaves but occasionally infesting other green (assimilatory) organs such as young bark, stems, buds, and maple fruits (samaras) [3,15]. Inhabiting the tissue beneath the epidermis, they create either slender to wide gallery-like or blotch-like mines, or a combination of both—a slender gallery in the early stage transitioning to a blotch in the final part. On occasion, Nepticulidae larvae induce galls in a leaf petiole [16] or leaf blade [17]. For in-depth insights into Nepticulidae biology, we recommend the following publications [3,18] including recent papers by Remeikis et al. [19] and Stonis et al. [6,15].

The larvae of Nepticulidae are obligatory miners, i.e., they feed inside plant tissues throughout all instars. A notable trophic characteristic of Nepticulidae is their narrow diet breadth (stenophagy). The majority of Nepticulidae species are either monophagous or strictly oligophagous, with occasional occurrences of broad oligophagy and disjunct oligophagy (see definitions provided in [6,15]).

While mining, the larvae not only consume part of a leaf’s photosynthetic tissue but also can damage leaf veins, resulting in significant harm. Despite adult pygmy moths being among the world’s smallest lepidopterans [20], their tiny larvae produce leaf mines that are notably large and conspicuous. These endobiotic insects, residing within plant tissues, are a natural component of nearly every terrestrial ecosystem and hold scientific interest for understanding general evolutionary processes. However, viewed from a consumer’s perspective rather than an ecological standpoint, Nepticulidae are often considered as plant pests or potential pests. Nepticulidae species have been listed as pests of cultivated plants [21], and as insects of concern in other surveys addressing pests [22,23]. While Nepticulidae leaf mines are typically not abundant, a significant population increase can lead to severe damage to the host plant. A notable infestation was reported in the Russian Far East [24], where 400–500 leaf mines of *Ectoedemia picturata* Puplesis on a single compound leaf of a cultivated *Rosa rugosa* Thunb caused the foliage to turn brown. The hips of this rose are a source of vitamin C [15]. Field observations indicate that some Palearctic species mines can induce premature leaf abscission, leading to defoliation and reduced fruit yield, e.g., [15,25,26]. A recent discovery highlighted a new pest of guava (*Psidium guajava* L.) in Colombia, South America, a significant tree cultivated for its fruit in local and international cuisine and traditional medicine [22].

**Figure 1:**
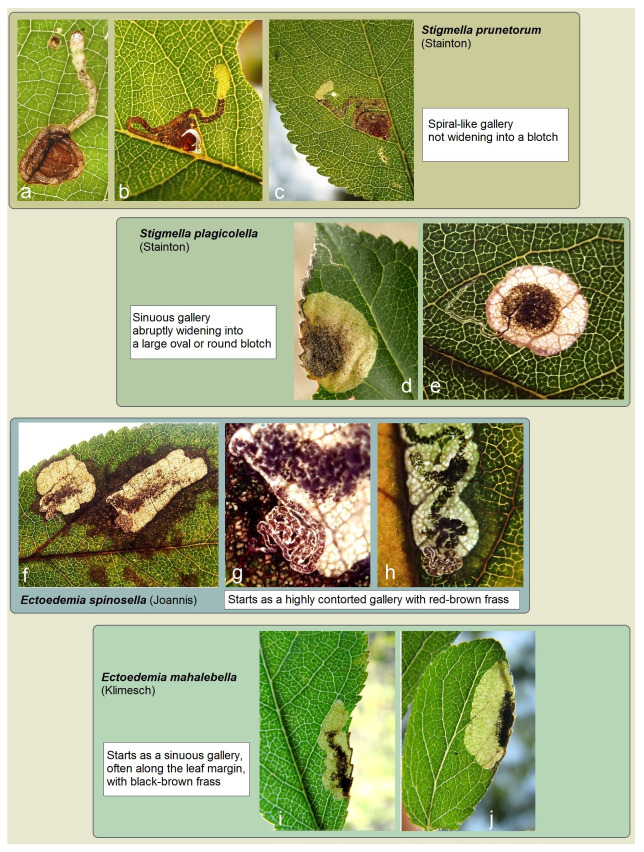
Diagnostics of the leaf mines of Nepticulidae species infesting plum trees in Europe: (**a**) *Stigmella prunetorum* (Stainton), Austria; (**b**) the same, Lithuania; (**c**) the same, Crimea; (**d**) *S. plagicolella* (Stainton), Austria; (**e**) the same, Bulgaria: Varna; (**f**) *Ectoedemia spinosella* (Joannis); (**g**,**h**) the same, Croatia, Zagreb; and (**i**,**j**) *E. mahalebella* (Klimesh), Crimea.

However, Nepticulidae, like other tiny endobiotic organisms, remain undersampled and understudied in many regions of the world. In Europe, which is the most extensively studied region concerning Nepticulidae, certain species of these moths infest plum trees (*Prunus* spp.). With the exception of a limited number of species that exhibit oligophagous behavior and may sporadically also feed on *Prunus*, Europe is home to four Nepticulidae species whose larvae are specialized to infest plum trees. All these species are characterized as leaf miners. Two belong to the genus *Stigmella* Schrank—*S. prunetorum* (Stainton) and *S. plagicolella* (Stainton)—while the other two belong to the genus *Ectoedemia* Busck—*E. spinosella* (Joannis) and *E. mahalebella* (Klimesch). Comprehensive documentation and illustrations of their adult external morphology and diagnostic characters of male and female genitalia already exist [3,16,27], etc. Despite this wealth of information, differentiation based solely on leaf mine samples can still be occasionally perplexing, especially when relying on internet sources. Therefore, we have included examples of leaf mines from all four species collected by us in Europe, accompanied by short differential notes highlighting diagnostic characteristics (Figure 1).

The Nepticulidae that feed on *Prunus* in the Caucasus, a vast region bordering Europe, remain insufficiently explored. It was anticipated that European *Prunus*-mining species might be widespread from Europe to the Caucasus and other regions of Western Asia. Indeed, the previously exclusive European species *Ectoedemia spinosella* was recently confirmed in the fauna of Turkmenistan and Tajikistan [14]. This year, *E. mahalebella* was reliably identified in the fauna of Armenia [28]. Among the most morphologically distinctive and well-known Nepticulidae species in Europe is *S. plagicolella*. Its occurrence in the Crimea, a peninsula separated from the Caucasus by the Kerch Strait and only 300–500 km away, has been reliably documented [29], suggesting the logical possibility of *S. plagicolella* existing in the Caucasus. The European *S. plagicolella* was previously mentioned in the Caucasus based on leaf mines in a monograph on the Eastern European and Asian Nepticulidae [11]. Furthermore, the recently published catalog of the Lepidoptera of Iran [30] also included records of the European *S. plagicolella* in Iran, specifically in localities bordering the southern regions of the Caucasus, such as Mazandaran, Golestan, and Gharbi.

During our recent fieldwork in the Caucasus, we observed leaf mines infesting various native and cultivated plum trees. Initially, these mines bore a striking resemblance to those caused by *S. plagicolella* in Europe. However, upon rearing the adults, we noted significant differences compared to the European *S. plagicolella*. Consequently, we hypothesized that our findings in the Caucasus are not attributable to *S. plagicolella*, challenging the accuracy of all previous records of *S. plagicolella* in Western Asia, including the most recent report from Iran [30].

To substantiate our hypothesis and distinguish the Caucasian *Prunus*-feeding Nepticulidae species from *S. plagicolella*, we expanded our fieldwork in the Caucasus. The aim was to acquire fresh material for molecular studies, gather more comparable data on species morphology and distribution, and collect, analyze, and document extensive leaf- mine samples. In parallel, the European *S. plagicolella* underwent re-examination. Originally described by Henry Tibbats Stainton in 1854 from southern London [31], the type material, housed at the Natural History Museum, London, consists of a single female. Our morphological examination of the type specimen prompted the collection of fresh *S. plagicolella* material from the type locality (London) and additional specimens from southern Europe (Croatia and Bulgaria) extending westward to the Black Sea and the Caucasus. We also re-examined *S. plagicolella* from the Crimea, a peninsula adjacent to the Caucasus.

The objective of this paper is to showcase the outcomes of our morphological, molecular, and statistical investigations, unveiling a previously unknown *Prunus*-feeding Nepticulidae species in the Caucasus. Our study, which unexpectedly identified a new species in the region, serves not only to document a potential new pest and enhance understanding of the Caucasian fauna but also to contribute to the broader biological inventory. Furthermore, we expect that our findings might encourage further research in Western Asia, with this also encompassing the Caucasus region.

## 2. Materials and Methods

Materials were obtained by collecting mined leaves from various plum trees (*Prunus* spp.) and rearing adults from the feeding larvae (the method is described in the following papers [6,11,13]). The material of *Stigmella colchica* sp. nov. became available through our fieldwork in the Caucasus in 2022–2023 and will be deposited at the MfN, Berlin, following this study. Additionally, specimens collected by Aleksey Maksimovich Gerasimov in Sochi (Russia) in 1932–1933 and by Rimantas Puplesis in Abkhazia, Sukhumi, and Novyy Afon (Georgia) in 1986 were available for our study (the type series will be transferred to the MfN, Berlin, after this study). Materials of the European *S. plagicolella* were obtained through our collecting efforts in 2023 in the type locality of *S. plagicolella* (London, Great Britain), as well as in Croatia and Bulgaria. Furthermore, some specimens previously collected by Arūnas Diškus in Lithuania and by Asta Navickaitė and Arūnas Diškus in the Crimea were available for our study from the BRG collection (this material will be transferred to the MfN, Berlin, after this study). The female of *S. plagicolella*, the only type specimen by Henry Tibbats Stainton (1822–1892), the author of *S. plagicolella*, deposited in the collection of the NHMUK (London, UK), was examined.

The methodologies and techniques for specimen preparation, species identification, and description were thoroughly detailed in previous works by Puplesis [11] and Stonis et al. [6]. Following the maceration of the abdomen in 10% KOH and subsequent cleaning, male genital capsules were extracted and mounted with the ventral side facing upward. The phallus was frequently removed and positioned alongside the genital armature using standard techniques [6]. Abdominal pelts and female genitalia were stained with Chlorazol Black (Direct Black 38/Azo Black), mounted in Euparal, and preserved throughout this study. Permanent preparations on microscope slides were photographed and examined using a Leica DM2500 microscope (Leica company, Wetzlar, Germany) equipped with a Leica DFC420 digital camera. Adult measurements and examinations were conducted using a Lomo stereoscopic microscope MBS-10 (Lomo company, St. Petersburg, Russia), and images were captured with a Leica S6D stereoscopic microscope (Leica company, Wetzlar, Germany) equipped with a Leica DFC290 digital camera.

**Molecular studies.** Genomic DNA extraction, mtDNA CO1-5′ amplification, and PCR product purification were performed following Orlovskytė et al. [32]. Both DNA strands were sequenced in BaseClear B.V. (Leiden, The Netherlands) using the ABI 3730xl 96-capillary DNA analyzer (Applied Biosystems, Foster City, CA, USA).

Sequences were double-checked with the eye and edited, if necessary, with BioEdit v. 7.2.5 [33]. They were deposited in the NCBI GenBank database (www.ncbi.nlm.nih.gov/genbank, accessed on 7 February 2024) under the accession numbers PP318226–PP318265.

The pairwise distances as well as the best fit model of sequence evolution for phylogenetic study applying the maximum likelihood method and considering the lowest log-likelihood criterion score were determined using the MEGA v.7 software [34]. The maximum likelihood (ML) tree was based on a GTR+G+I model and 10,000 bootstrap replications implemented in MEGA. The Bayesian phylogenetic analysis was carried out using the Markov chain Monte Carlo (MCMC) method with MrBayes v. 3.2.3 [35], under a GTR+G+I model and run for 5 million generations, with tree sampling every 10,000 generations. Species delimitation was estimated using the Assemble Species using Automatic Partitioning (ASAP) (bioinfo.mnhn.fr/abi/public/asap/asapweb.html, accessed on 7 February 2024) [36] and the Bayesian Poisson Tree Processes method (bPTP) (species.h-its.org/ptp/, accessed on 7 February 2024) [37]. ClustalX2 [38] was used for the conversion of the Fasta format to Nexus. Genetic diversity parameters were estimated with the DnaSP v.6 software [39]. The mitotype network was constructed with the Templeton, Crandall, and Sing (TCS) Network algorithm [40] using the PopArt v.1.7 program (popart.otago.ac.nz, accessed on 7 February 2024).

**Statistical analysis.** The following criteria were counted and analyzed: (1) egg deposition and location on the host-plant leaf; (2) leaf-mine location (apical, median, or basal placement on the leaf blade, also marginal or not marginal, i.e., inner, not reaching the margin of the leaf); and (3) leaf-mine shape (regular or irregular).

To analyze differences in egg deposition, leaf-mine location, and leaf-mine morphology, we used pooled samples, and traits were expressed as proportions (in percent), with 95% confidence intervals (CI), calculated as Wilson’s CI [41]. The Wilson method is accurate, suitable for small samples and provides better coverage of confidence intervals, especially when dealing with extreme proportions [42]. The significance of differences between observed numbers was evaluated with chi-square, and those of proportions with a G-test. Comparing numbers, observed frequencies were proportionally realigned to the same sample size. Graphs were completed in MS Excel, calculations were completed in Past and using a G-test online calculator [43].

**Abbreviations for institutions and specimen depositories**: BRG—Biosystematic Research Group, currently based at the State Research Institute Nature Research Centre, Vilnius, Lithuania; MfN—Museum für Naturkunde, Berlin, formerly known as the Museum der Naturkunde für Humboldt Universität zu Berlin or Museum für Naturkunde/Leibniz-Institut für Evolutions und Biodiversitätsforschung, Berlin, Germany; NHMUK (formerly BMNH)—The Natural History Museum, London, Great Britain.

## 3. Results

### 3.1. Taxonomic Account: A Description of a New Species

***Stigmella colchica* Stonis & Diškus, sp. nov.**

urn:lsid:zoobank.org:act:81EE3626-175E-40F2-98CD-3EB65F4A525E

**Figure 2:**
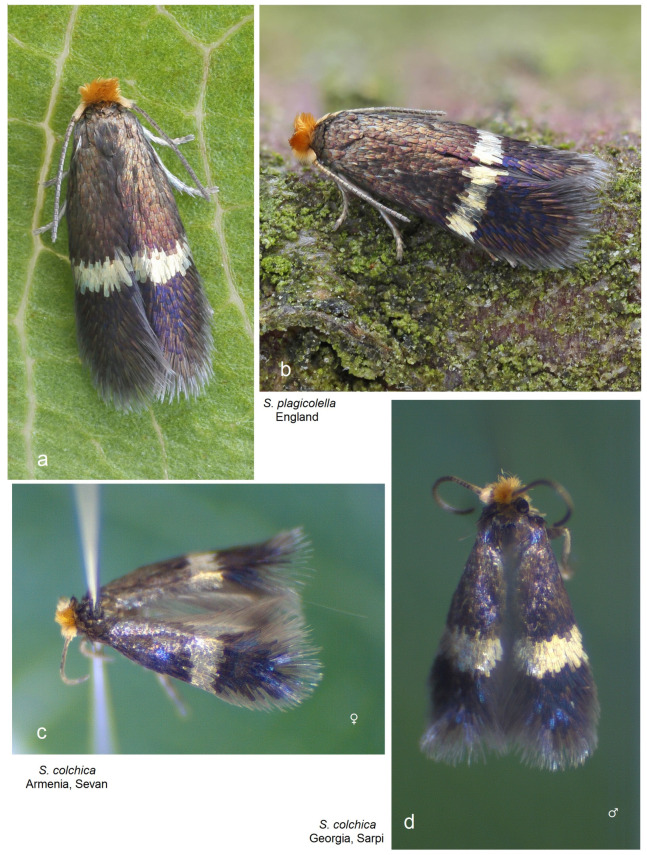
Adults: (**a**,**b**) *Stigmella plagicolella* (Stainton), England (courtesy of Patrick Clement, United Kingdom); (**c**) *S. colchica* sp. nov., Armenia; and (**d**) the same, Georgia.

**Figure 3:**
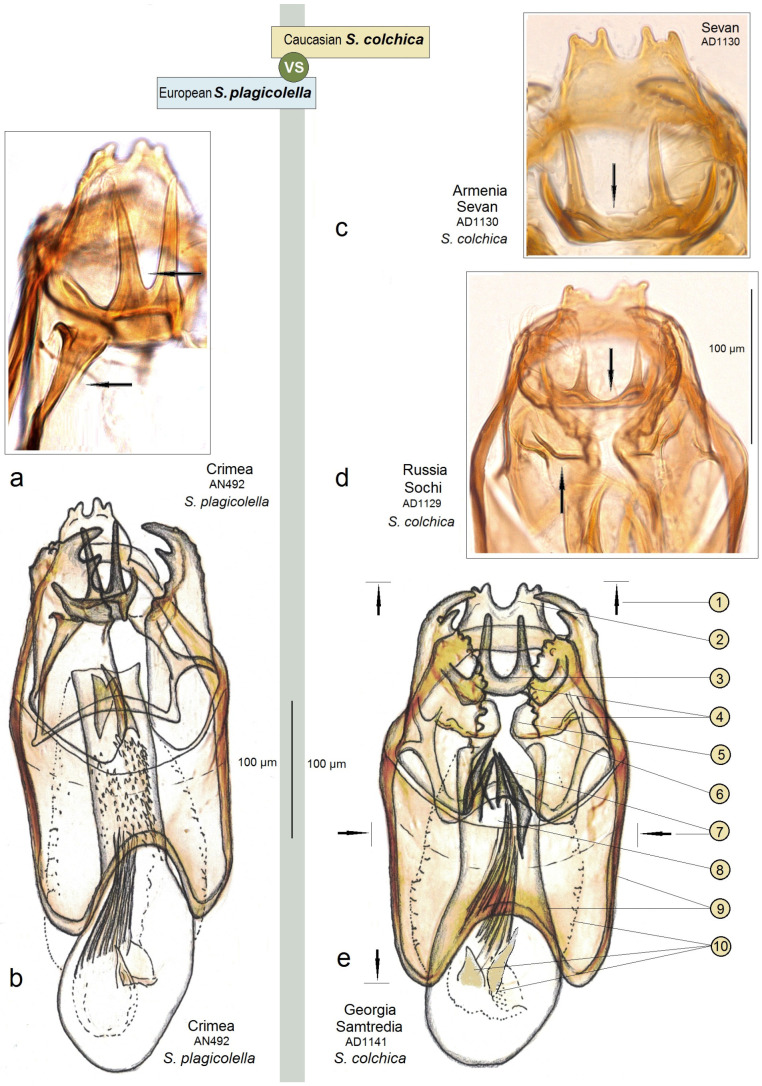
Differentiation based on the characters of the male genitalia. The Caucasian *Stigmella colchica* sp. nov. vs. the European *S. plagicolella* (Stainton): (**a**,**b**) *S. plagicolella*, and (**c**–**e**) *S. colchica* sp. nov. (Note the diagnostic characteristics 1–10; the arrows highlight the different sizes).

**Figure 4:**
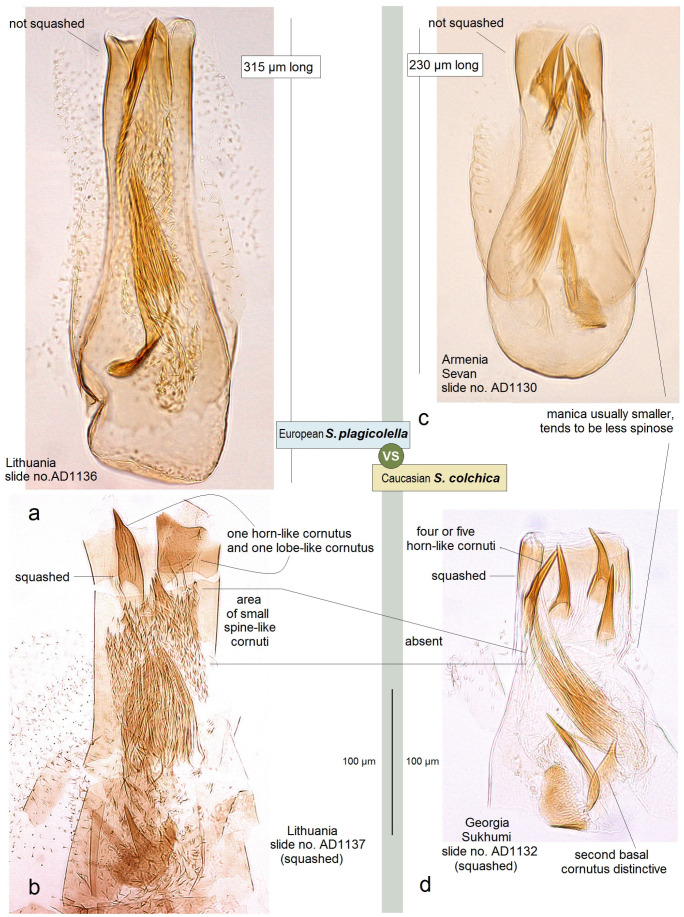
Differentiation based on the characters of the phallus. The Caucasian *Stigmella colchica* sp. nov. vs. the European *S. plagicolella* (Stainton): (**a,b**) *S. plagicolella*; and (**c**,**d**) *S. colchica* sp. nov.

**Diagnosis.** This new species belongs to the *Stigmella sorbi* species group. *Stigmella colchica* sp. nov. is most similar and closely related to the European *S. plagicolella* (Stainton). Externally, this new species can be confused with *S. plagicolella* (Figure 2). In the male genitalia, *S. colchica* sp. nov. can be distinguished from *S. plagicolella* by the U-shaped gnathos without anterior processes (v-shaped, with anterior lobe-like processes in *S. plagicolella*), the presence of four distinctive, spine-like apical cornuti, the long basal cluster of slender cornuti, and the absence of scalop-like cornuti. Additionally, it differs from *S. plagicolella* by the shorter but wider phallus, the medially distinctly bulged valva, and the wider but usually shorter genitalia capsule (slender and long in *S. plagicolella*) (Figure 3 and Figure 4; also see characters 1–10 in Figure 3). In the female genitalia, *S. colchica* sp. nov. resembles *S. plagicolella*, and only slightly differs by the caudally bulged corpus bursae, folded vestibulum, and more elaborated ovipositor.

**Figure 5:**
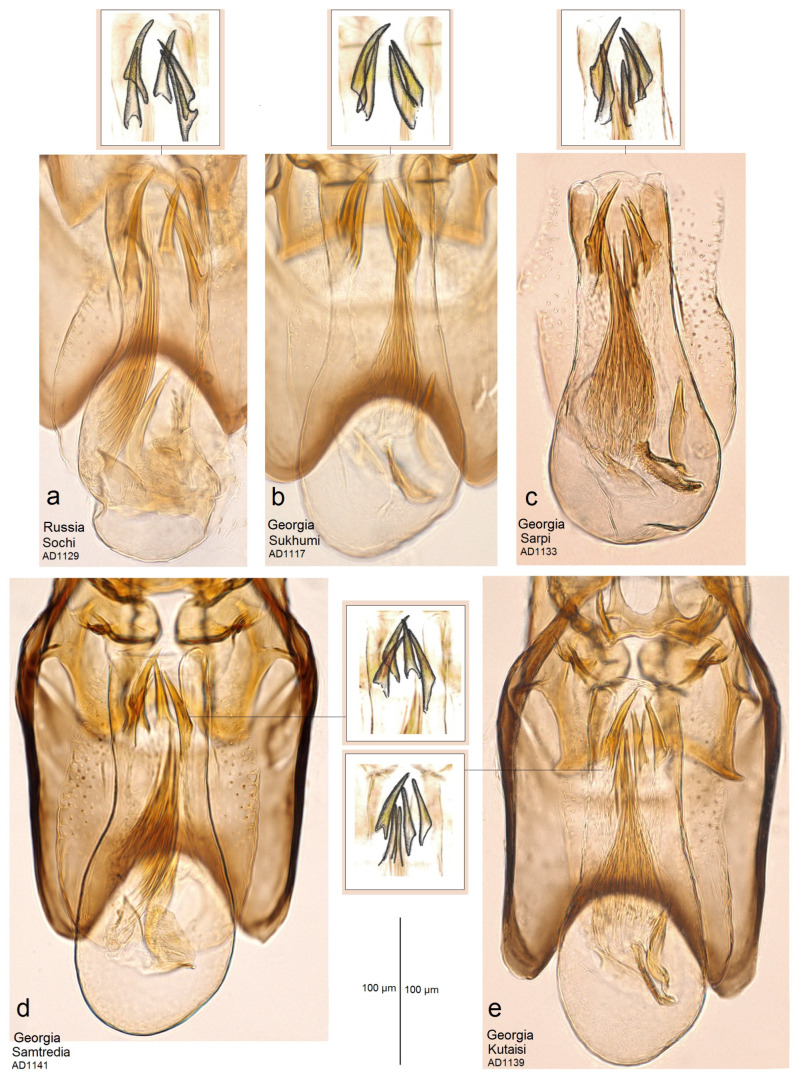
Phallus of *Stigmella colchica* sp. nov.: (**a**) genitalia slide AD1129, Sochi, Russia; (**b**) slide AD1117, Sukhumi, Georgia; (**c**) slide AD1133, Sarpi, Georgia; (**d**) slide AD1141, Samtredia, Georgia; and (**e**) slide AD1139, Kutaisi, Georgia (MfN).

**Figure 6:**
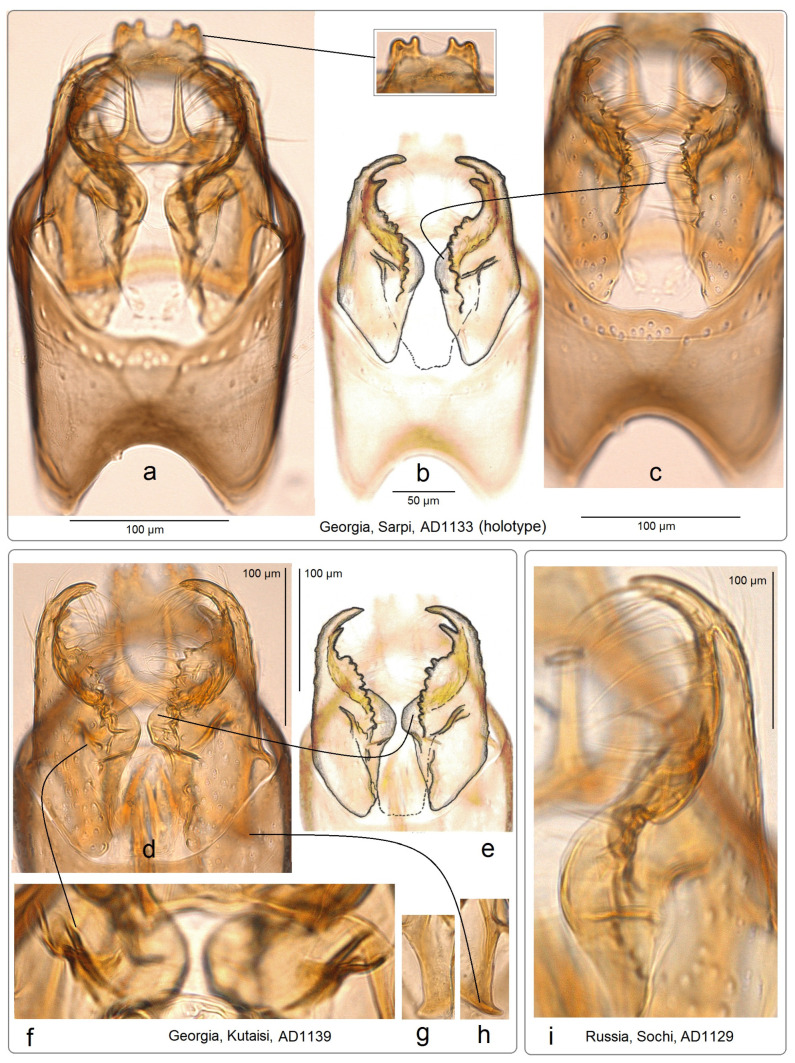
Details of the genitalia of *Stigmella colchica* sp. nov.: (**a**–**c**) capsule, genitalia slide AD1133, Sarpi, Georgia; (**d**–**h**) inner bulge and thickening of the valva and transtilla, genitalia slide AD1139, Kutaisi, Georgia; and (**i**) valva, genitalia slide AD1139, Sochi, Russia (MfN).

**Male:** The forewing length is 1.6–1.9 mm; the wingspan is 3.6–4.2 mm (*n* = 15). Head: The palpi are cream to dark golden cream; the frontal tuft is beige–orange to dark orange; the collar is fuscous, with purple iridescence; the scape is glossy golden cream to dark golden cream; the antenna is significantly shorter than half the length of the forewing, with 28–29 segments; the flagellum is glossy grey to fuscous, with some purple iridescence on the upper side, pale grey on the underside; occasionally, the flagellum is glossy pale golden grey. Thorax: The tegula, thorax and the forewing are densely covered by brown–black scales with strong purple iridescense (especially intense apically); the fascia is slightly postmedian, wide, shiny golden; the fringe is grey–black, pale-grey distally, without a fringe line; the forewing underside is blackish–grey, without spots or androconia. The hindwing and its fringe are grey to dark grey with light-golden gloss, without androconia. The legs are glossy blackish-grey to golden grey, with some purple iridescence. Abdomen: On the upper side and underside, the abdomen is brown–black with some purple iridescence. The genitalia capsule (Figure 5 and Figure 6) is 305–350 µm long, 185–195 µm wide. The tegumen is band-shaped. The uncus has four distal papillae. The gnathos is U-shaped, with two well-separated caudal processes but without anterior lobes. The valva has two apical processes (long apical and short subapical process) and an inner bulge medially. The vinculum is relatively wide (wider and usually shorter than in the related *S. plagicolella*), with two large lateral lobes. The phallus is 230–280 µm long (wider and shorter than in the related *S. plagicolella*), with four to five large spine-like cornuti apically, numerous slender cornuti collected in a very long cluster, two basal cornuti, but without scalop-like cornuti medially (which are characteristic of the related *S. plagicolella*).

**Figure 7:**
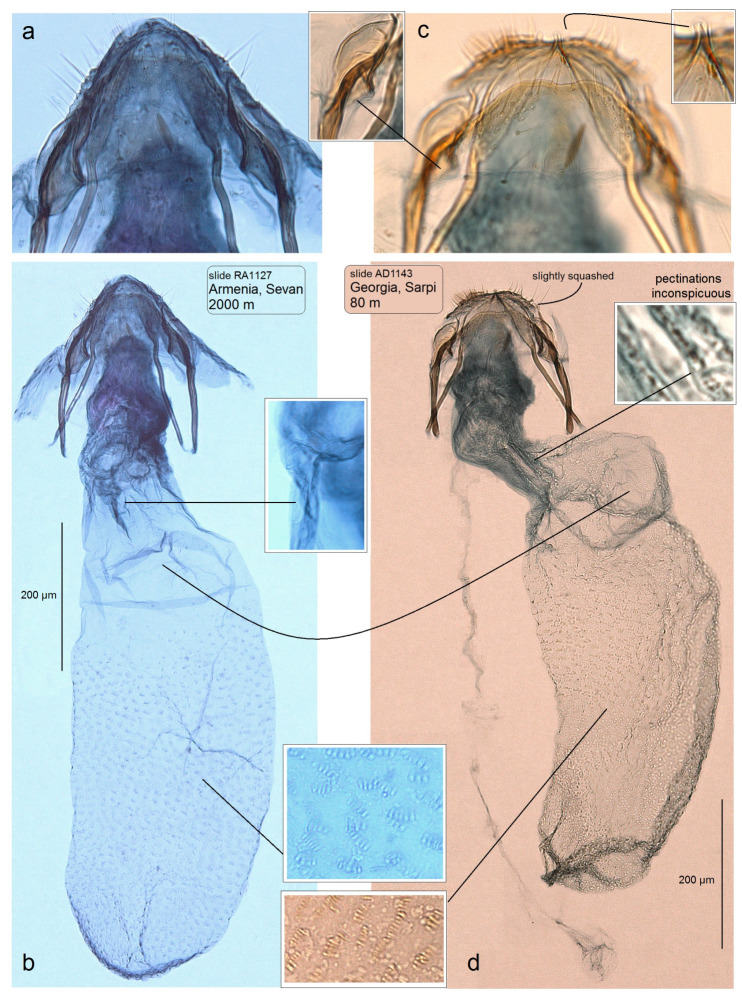
Female genitalia of *Stigmella colchica* sp. nov.: (**a**,**b**) genitalia slide RA1127, Sevan, Armenia, and (**c**,**d**) genitalia slide AD1143, Sarpi, Georgia (MfN).

**Female:** The forewing length is 1.9–2.0 mm; the wingspan is 4.3–4.5 mm (*n* = 10). Similar to the male, but the antenna is significantly shorter than one-third the length of forewing, it has 20 segments. The legs are glossy and dark grey, with purple iridescence. Abdomen: On the upper side and underside, the abdomen is brown–black with some purple iridescence. The genitalia (Figure 7) is 860–1045 µm long. The abdominal apex is triangular or rounded, with short setae; the anterior and posterior apophyses are equal in length. The vestibulum is strongly folded, without a vaginal sclerite or distinctive pectinations (at least obvious pectinations). The corpus bursae is elongated, densely covered with pectinations, except for a bulged proximal part. The ductus spermathaecae is without coils, and slightly sinuous.

**Figure 8:**
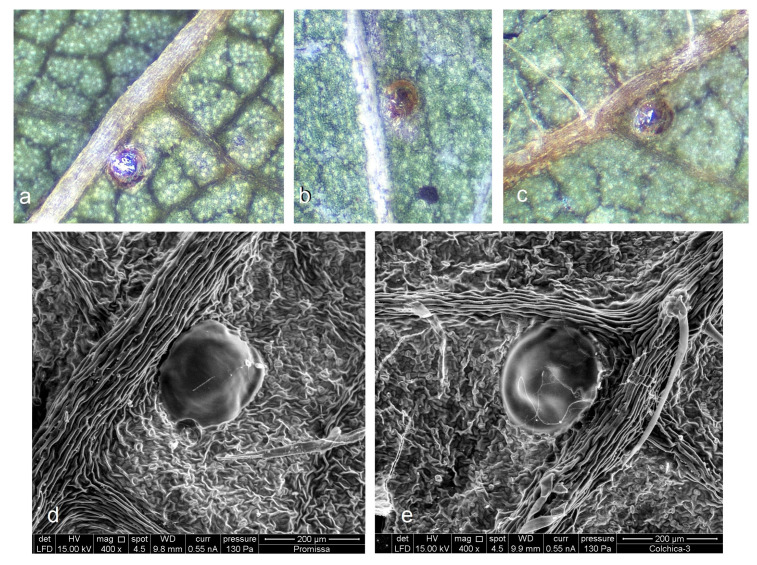
Egg case of *Stigmella colchica* sp. nov., Georgia: (**a**–**c**) documented using a Leica S6D stereoscopic microscope with an attached Leica DFC290 digital camera, and (**d**,**e**) photography performed with a scanning electron microscope FEI Quanta 250.

**Figure 9:**
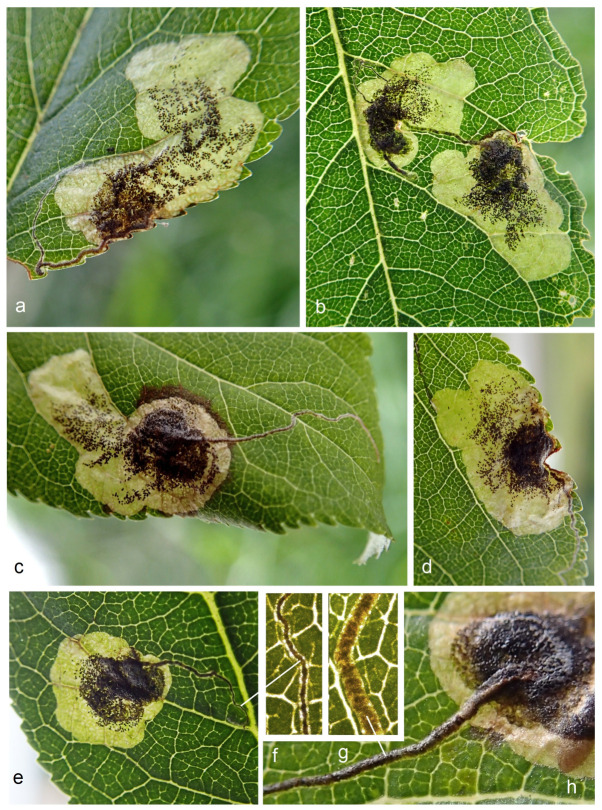
Leaf mines of *Stigmella colchica* sp. nov., Georgia: (**a**–**e**) general view of the leaf mine, and (**f**–**h**) slender initial part of the leaf mine filled with frass.

**Figure 10:**
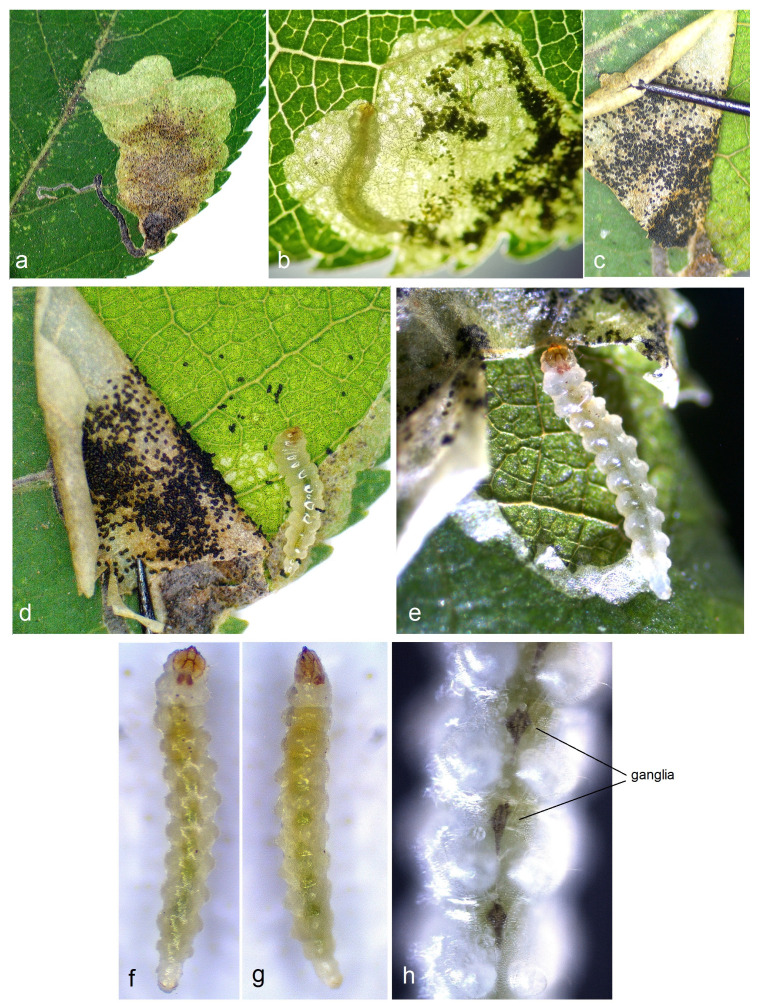
Bionomics of *Stigmella colchica* sp. nov., Georgia: (**a**) a view of the leaf mine in natural illumination (without using transmitted light); (**b**–**e**) leaf mine with the upper epidermis opened (torn); and (**f**–**h**) larva.

**Bionomics** (Figure 8, Figure 9 and Figure 10). The host plants are *Prunus divaricata* Ledeb., *P. cerasifera* Ehrh., and various cultivated plum trees; occasionally, leaf mines of *S. colchica* sp. nov. were also discovered on *Cerasus* sp. The egg is laid on the underside of the leaf, sometimes on the upper side; the egg case is rounded, glossy. The larvae usually mine leaves from July to early August, but can continue mining from September to early October in some lowland areas. Judging from observed old (empty) leaf mines, mining activities also occur in June. The larva is pale yellowish-white with a brownish-green intestine and a pale-brown head. The leaf mine is a combination of a slender gallery and an abruptly widening blotch with black frass; the frass is not loose, but adheres to the upper epidermis. The cocoon is dark yellowish–beige, oval-shaped, measuring 1.8–2.4 mm in length and 1.8–2.5 mm in width. Adults are known from July to September, and after pupal hibernation in cocoons, they occurred in April–May (or February if hibernated pupae in cocoons were reactivated indoors).

**Figure 11:**
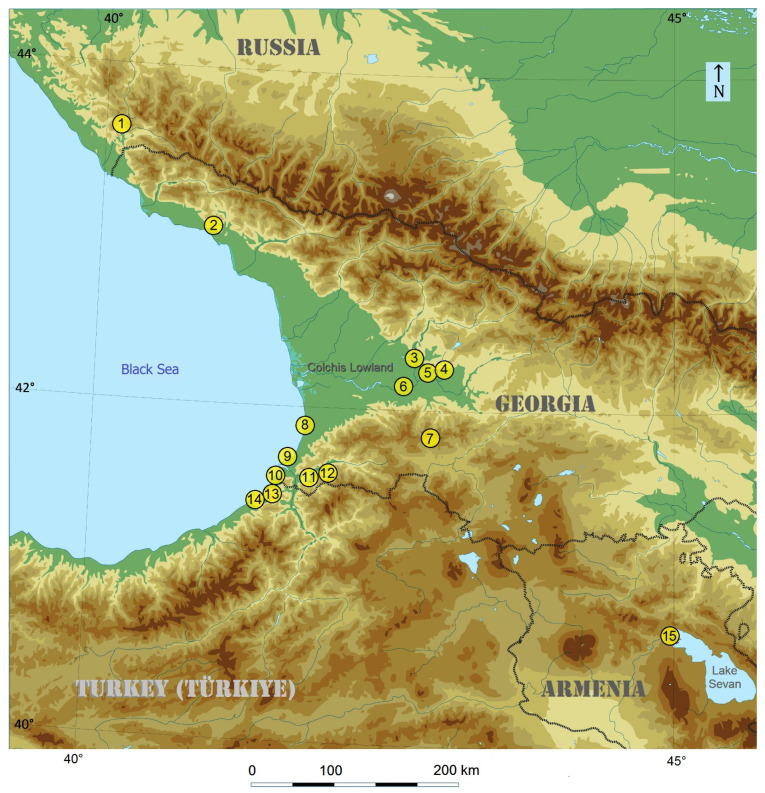
The geographical distribution of *Stigmella colchica* sp. nov. detected during the current study (1—Krasnaya Polyana, Sochi, Krasnodar Krai, Russia, 43°40′18″ N, 40°11′37″ E, ca. 540 m; 2—Akhali Atoni (=Novyi Afon), Abkhazia, Georgia, 43°5′6″ N, 40°48′24″ E, ca. 40 m; 3—Tskhaltubo, Georgia, 42°18′59″ N, 42°35′24″ E; 4—Motsameta, Georgia, 42°16′59″ N, 42°45′18″ E; 5—Kutaisi, Georgia, 42°16′32″ N, 42°42′36″ E; 6—Samtredia, Georgia, 42°9′47″ N, 42°20′34″ E; 7—Sairme, 41°54′19″ N, 42°44′56″ E, ca. 1050 m; 8—Kobuleti, Adjara AR, Georgia, 41°49′47″ N, 41°46′43″ E, ca. 10 m; 9—Batumi, N of Botanical Garden, Adjara AR, Georgia, 41°42′38″ N, 41°43′42″ E, ca. 25 m; 10—Sarpi, Adjara AR, Georgia, 41°31′26″ N, 41°33′2″ E, ca. 80 m; 11—SE of Batumi, Mirveti, Adjara AR, Georgia, 41°31′36″ N, 41°42′58″ E, 150 m; 12—Zeda Chkhutuneti, Machakhela N.P., Adjara AR, Georgia, 41°29′5″ N, 41°51′28″ E, ca. 600 m; 13—Üçkardeş, Artvin Province, Karadeniz Bölgesi, Türkiye (=formerly, Turkey), 41°29′35″ N, 41°32′24″ E, 70 m; 14—Hopa, Artvin Province, Karadeniz Bölgesi, Türkiye 41°23′39″ N, 41°25′2″ E, ca. 5 m; and 15—Sevan (Tsovagyugh), Armenia, 40°36′8″ N, 44°57′45″ E, ca. 2000 m (note that the type locality of *S. colchica* sp. nov. is shown under no. 10).

**Figure 12:**
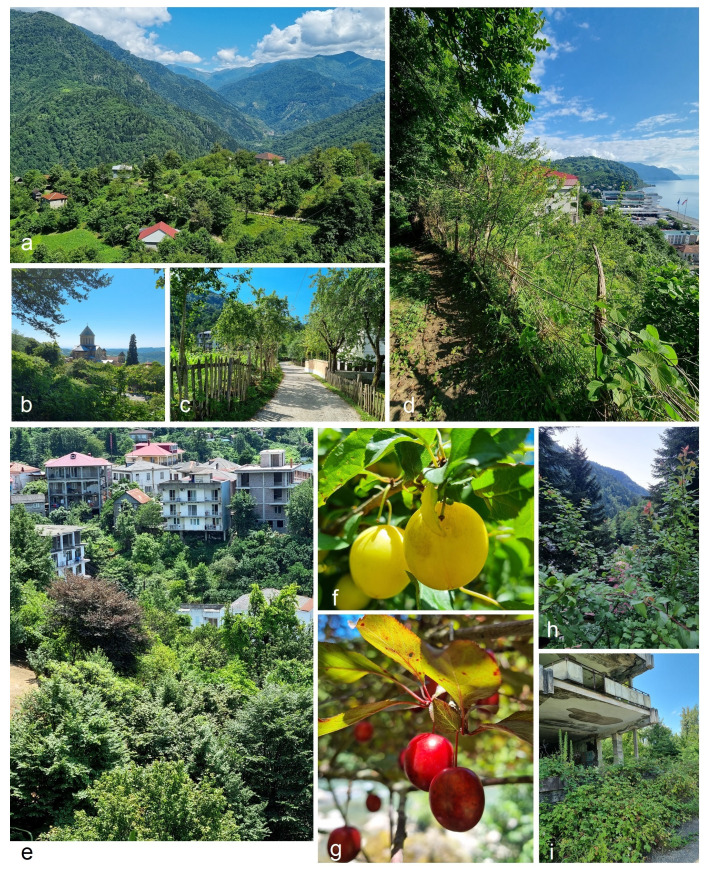
(**a**–**i**) *Stigmella colchica* sp. nov. inhabits various habitats of moist subtropical forest of the Colchis Lowland at altitudes ranging from 5 m to 100 m and, in the valleys of mountainous areas, from 600 to 2000 m, but appears to be most abundant in disturbed or cultivated areas, including orchards.

**Distribution** (Figure 11). The species is widespread and common along the eastern coast of the Black Sea, including southern Russia (Sochi), Georgia (Sukhumi, Novyy Afon, Kobuleti, Batumi, Sarpi), and Türkiye (Karadeniz Bölgesi: Üçkardeş and Hopa). It is also common in the Colchis Lowland (Georgia) and the lower slopes of the northern and southern Caucasus. In Armenia, this new species is known to occur even at an elevation of 2000 m (Sevan: Tsovagyugh). The recent records of *S. plagicolella* in Iran [30] may also pertain to the new species, *S. colchica* sp. nov. The latter was found to be common in various localities of the moist subtropical forest of the Colchis Lowland at altitudes ranging from 5 m to 100 m and in the valleys of mountainous areas from 600 m to 2000 m. *S. colchica* sp. nov. appears to be common and most abundant in disturbed or cultivated areas including orchards (Figure 12).

**Etymology.** The species is named after the Colchis Lowland in Georgia, referring to the locality where the majority of specimens in the type series were collected.

**Type material.** Holotype: ♂, GEORGIA: Adjara AR, Sarpi, 41°31′26″ N, 41°33′2″ E, ca. 80 m, mining larvae on *Prunus* sp., 19–20 July 2023, leg. S.R. Hill & J.R. Stonis, genitalia slide no. AD1133♂ (MfN). Paratypes (20 ♂, 21 ♀): 9 ♂, 9 ♀, same label data as holotype, genitalia slide nos AD1142♂, AD1143♀ (MfN); 1 ♂, Adjara AR, Batumi, N of Botanical Garden, 41°42′38″ N, 41°43′42″ E, ca. 25 m, 17 July 2023, leg. J.R. Stonis; 1 ♀, Adjara AR, Kobuleti, 41°49′47″ N, 41°46′43″ E, ca. 10 m, 23 July 2023, leg. S.R. Hill & J.R. Stonis; 1 ♂, Abkhazia: Akhali Atoni (Novyi Afon), 43°5′6″ N, 40°48′24″ E, ca. 40 m, mining larvae on *Prunus* sp. 26 October 1986, ex pupa indoors 10 February1987, leg. R. Puplesis, genitalia slide no. AD1117♂ (MfN); 1 ♂, 1 ♀, Abkhazia, Sukhumi, 41°38′11″ N, 41°39′0″ E, ca. 90 m, mining larvae on *Prunus* sp. 26 October 1986, ex pupa indoors 10 February1987, leg. R. Puplesis, genitalia slide no. AD1132♂ (MfN); 5 ♂, 5 ♀, Kutaisi, 42°16′32″ N, 42°42′36″ E–42°17′3″ N, 42°43′42″ E, 25 July 2023, leg. S.R. Hill & J.R. Stonis, genitalia slide no. AD1139♂ (MfN); 1 ♂, 2 ♀, Samtredia, 42°9′47″ N, 42°20′34″ E, 24 July 2023, leg. S.R. Hill & J.R. Stonis, genitalia slide no. AD1141♂ (MfN); 1 ♀, Sairme, 41°54′19″ N, 42°44′56″ E, ca. 1050 m, 15 July 2023, leg. J.R. Stonis; 1 ♂, 1 ♀, ARMENIA: Sevan (Tsovagyugh), 40°36′8″ N, 44°57′45″ E, 2000 m, larva on *P. divaricata* Ledeb., 9 August 2022, leg. J.R. Stonis, genitalia slides nos AD1130♂, RA1127♀ (MfN); 1 ♀, RUSSIA: Krasnaya Polyana, Sochi, Krasnodar Krai, 43°40′18″ N, 40°11′37″ E, ca. 540 m, mining larvae on *Prunus* sp. 22 September 1932, leg. A.M. Gerasimov; 1 ♂, 1 ♀, same locality as previous paratype, 24 April 1933, leg. A.M. Gerasimov, genitalia slide no. AD1129♂ (MfN).

**Other studied material.** GEORGIA: 4 larvae (consumed for DNA studies) and leaf-mine sample on *Prunus* sp., Motsameta, 42°16′59″ N, 42°45′18″ E, 14 July 2022, leg. J.R. Stonis (BRG); 3 larvae and 3 adults of unknown sex (consumed for DNA studies), on Prunus sp., Kutaisi, 42°16′32″ N, 42°42′36″ E–42°17′3″ N, 42°43′42″ E, 25 July 2023, leg. S.R. Hill & J.R. Stonis; leaf-mine sample, Kutaisi, 42°16′28″ N, 42°42′39″ E, 25 July 2023, old leaf mines on *Cerasus* sp., leg. J.R. Stonis (BRG); leaf-mine sample, Tskhaltubo, 42°18′59″ N, 42°35′24″ E, on *Prunus* sp., 15 July 2023 and 25 July 2023, leg. J.R. Stonis (BRG); 4 adults of unknown sex (consumed for DNA studies), on *Prunus* sp., Samtredia, 42°9′47″ N, 42°20′34″ E, 24 July 2023, leg. S.R. Hill & J.R. Stonis; 1 ♀ adult (consumed for DNA studies), on *Prunus* sp., Sairme, 41°54′19″ N, 42°44′56″ E, ca. 1050 m, 15 July 2023, leg. J.R. Stonis; 1 ♀ adult (consumed for DNA studies), on *Prunus* sp., Adjara AR, Kobuleti, 41°49′47″ N, 41°46′43″ E, ca. 10 m, 23 July 2023, leg. S.R. Hill & J.R. Stonis; leaf-mine sample, on *Prunus* sp., Batumi (city), 41°38′21″ N, 41°37′44″ E, ca. 25 m, 23 July 2023, leg. S.R. Hill & J.R. Stonis (BRG); 1 ♀ adult (consumed for DNA studies), on *Prunus* sp., Adjara AR, Batumi, N of Botanical Garden, 41°42′38″ N, 41°43′42″ E, ca. 25 m, 17 July 2023, leg. J.R. Stonis; leaf-mine sample, on *Prunus* sp., Adjara AR, SE of Batumi, Mirveti, 41°31′36″ N, 41°42′58″ E, ca. 150 m, 18 July 2023, leg. J.R. Stonis (BRG); leaf-mine sample, on *Prunus* sp., Adjara AR, Machakhela N.P., Zeda Chkhutuneti, 41°29′5″ N, 41°51′28″ E, ca. 600 m, 18. July 2023, leg. J.R. Stonis (BRG); 2 larvae and 5 adults of unknown sex (consumed for DNA studies), on *Prunus* sp., Adjara AR, Sarpi, 41°31′26″ N, 41°33′2″ E, ca. 80 m, mining larvae on *Prunus* sp., 19–20 July 2023, leg. S.R. Hill & J.R. Stonis.

TÜRKIYE: 3 larvae (consumed for DNA studies) and leaf-mine sample, on cultivated *Prunus* sp., Karadeniz Bölgesi, Artvin Province, Üçkardeş, 41°29′35″ N, 41°32′24″ E, 70 m, 20 July 2023, leg. S.R. Hill & J.R. Stonis (BRG); 1 larva (consumed for DNA studies) and leaf-mine sample, on cultivated *Prunus* sp., Karadeniz Bölgesi, Artvin Province, Hopa, 41°23′39″ N, 41°25′2″ E, ca. 5 m, on *Prunus cerasifera*, 20 July 2023, leg. J.R. Stonis & S.R. Hill (BRG).

### 3.2. Molecular Account: The Caucasian Stigmella colchica sp. nov. vs. the European S. plagicolella

The 674 base pairs (bp) long mtDNA CO1-5′ sequences of 30 *Stigmella colchica* sp. nov. and ten *S. plagicolella* specimens were successfully obtained. The current dataset with previous published sequences from the Barcode of Life Data System (BOLD) database v. 4 [44] (www.barcodinglife.org, accessed on 7 February 2024) includes 93 sequences of these two species (Table S1); *Pseudopostega cucullata* Stonis & Vargas (Opostegidae) was used as an outgroup.

**Figure 13:**
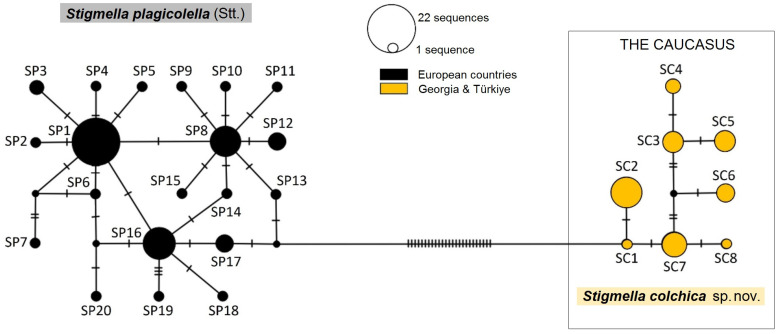
The mitotype network of the 588 bp long mtDNA CO1-5′ *Stigmella colchica* sp. nov. and *S. plagicolella* (Stainton) sequences constructed using the TCS Network algorithm. It includes 40 newly analyzed and 53 downloaded from BOLD sequences (Table S1). Each circle represents a unique mitotype; color indicates the region of specimen origin. The preliminary names of mitotypes are shown next to the circles; the size of each circle is proportional to mitotype frequency. The unnamed small circles represent mitotypes not sampled in the study but predicted to be. Dashes on the lines connecting mitotypes indicate hypothesized mutational steps.

According to the constructed TCS network based on the 588 bp long CO1-5′ sequences of 93 specimens of *S. colchica* sp. nov. and *S. plagicolella* (Figure 13), the mitotypes were clearly separated into two groups. One of them included the sequences of the specimens collected in European countries (*S. plagicolella* species), while the second one consisted of Georgian and Turkish sequences (*S. colchica* sp. nov.). They differ from each other by at least 26 hypothesized mutational steps, while within species, the mitotypes distinguish from each other with 7–8 mutational steps at most.

In total, 8 mitotypes were identified from the 30 analyzed *S. colchica* sp. nov. sequences (Table S1, Figure 13) defined using nine parsimony informative sites. The overall mitotype and nucleotide diversity was Hd = 0.85 ± 0.037 and π = 0.006 ± 0.0004, respectively. The neutrality indices Tajima’s D, Fu and Li‘s D, Fu and Li‘s F were not statistically significant. Possibly due to the limited number of sequences, *S. colchica* sp. nov. mitotypes did not form distinct groups. The most frequent mitotype, preliminary named after the species name as SC2, was the only mitotype, collected not only in Georgia, but also in Turkey; it was detected in nine specimens and accounted for 30% of all analyzed *S. colchica* sp. nov. sequences, and other mitotypes were rarer. The biggest difference between mitotypes was seven mutational steps (between SC2 and SC4). Georgia had the most diverse population observed in the present study, with eight mitotypes recorded.

The 63 *S. plagicolella* sequences were defined by six parsimony informative sites distributed to 20 mitotypes (Table S1, Figure 13). The diversity of mitotypes Hd reached 0.84 ± 0.035, while nucleotide diversity π was equal 0.003 ± 0.0003. All calculated neutrality indices were statistically significant: Tajima‘s D = -2.028 (*p* < 0.05), Fu and Li‘s D = −4.15, Fu and Li‘s F = −4.03 (both *p* < 0.02). The *S. plagicolella* mitotypes were clustered into three closely related groups with one or two mutational steps between each other. The major and most common mitotype was preliminarily named after the species name as SP1 (found in Bulgaria, Croatia, Denmark, and Lithuania), SP8 (collected in Bulgaria), and SP16 (from France, Germany, the Netherlands, and the United Kingdom). Among them, the frequency of SP1 was the highest, as it accounted for 34.92% of the total number of *S. plagicolella* sequences. SP8 and SP16 were also relatively common: they included 14.29% and 15.87%, respectively. The major groups were surrounded by other, peripheral, mitotypes, some of which consisted of only one sequence (e.g., SP14 from France, SP15 from Austria). The most different mitotypes among them appeared to be SP7 from Croatia and SP19 from Greece: they differed by eight mutational steps. One of the analyzed mitotypes, preliminarily named as SP7 and detected in Croatia, appeared to be a new one. The largest number of mitotypes (ten) was found in Bulgaria.

**Figure 14:**
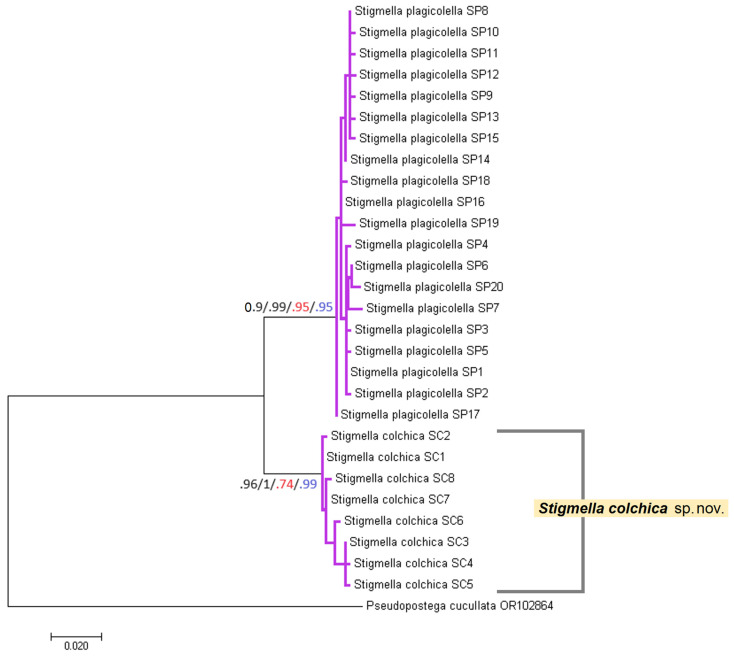
The phylogenetic relationships of *Stigmella colchica* sp. nov. and *S. plagicolella* reconstructed based on the 588 bp long mtDNA CO1-5‘ sequences and using GTR+G+I evolution model. Numbers of branches represent the bootstrap values obtained for maximum likelihood probability (10,000 bootstrap replicates) (black)/Bayesian posterior probability (5,000,000 generations) (black)/ASAP probability (red)/bPTP support value (blue). Magenta clusters of the tree were supported as separate species using both ASAP and bPTP delimitation algorithms. *Pseudopostega cucullata* was included as an outgroup.

The topologies of the maximum likelihood and the Bayesian inference based on the 588 bp long mtDNA CO1-5′ sequences available to us were mostly consistent (Figure 14). They reliably grouped the Caucasian *S. colchica* sp. nov. and European *S. plagicolella* specimens into two separate sister clades (*S. colchica* sp. nov. ML bootstrap value was equal to 96%, Bayesian posterior probability—100%; *S. plagicolella* ML—90%; Bayesian probability—99%), supported by both applied species delimitation methods (*S. colchica* sp. nov. ASAP—74%, bPTP—99%; *S. plagicolella* ASAP and bPTP—95% each). Comparison of estimates of evolutionary divergence between sequences supported the species status of *S. colchica* sp. nov. too; the pairwise distances of its sequences ranged from 0.15 ± 0.15% (e.g., between SC1 and SC2 mitotypes) to 1.22 ± 0.44% (e.g., between SC4 and SC5), while the divergence between *S. colchica* sp. nov. and *S. plagicolella* varied from 4.72 ± 0.95% (between SC1 and SP13 mitotypes) to 6.17 ± 1.05 (between SC8 and SP7).

### 3.3. Statistical Analysis of Leaf-Mine Samples

**Figure 15:**
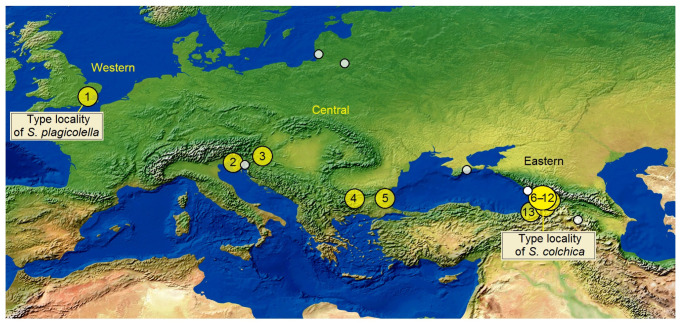
Locations of leaf-mine samples. Western: London, Great Britain (1); Central: Rovinj, Croatia (2), Zagreb, Croatia (3), Sofia, Bulgaria (4), Varna, Bulgaria (5); Eastern: Kutaisi, Georgia (6), Sairme, Georgia (7), Samtredia, Georgia (8), Kabuleti, Georgia (9), Batumi, Georgia (10), Mirveti and Machakhela N.P., Georgia (11), Sarpi, Georgia (12), Hopa and Üçkardeş, Türkiye (13). Note: the white dots show additional samples which were available, but not included in the statistical analysis because of their small size (Geographical map base, courtesy of T. Patterson, USA).

Sample locations and size: The following thirteen samples of leaf mines were collected, studied and included in the statistical analysis (yellow dots in Figure 15):

(1) *Stigmella plagicolella*, a sample of 246 leaf mines from GREAT BRITAIN, London (the type locality of *S. plagicolella*), 51°30′27.2″ N 0°25′39.3″ W, 8 August 2023, leg. J.R. Stonis & S.R. Hill;
(2) *S. plagicolella*, a sample of 52 leaf mines from CROATIA, Rovinj, 45°04′27.1″ N 13°39′05.2″ E, 11 August 2023, leg. J.R. Stonis & S.R. Hill;
(3) *S. plagicolella*, a sample of 318 leaf mines from CROATIA, Zagreb (Jarun), 45°46′38.9″ N 15°55′47.2″ E, 19–20 August 2023, leg. J.R. Stonis & S.R. Hill;
(4) *S. plagicolella*, a sample of 366 leaf mines from BULGARIA, Sofia, 42°40′40.7″ N 23°18′52.1″ E, 23 August 2023, leg. S.R. Hill & J.R. Stonis;
(5) *S. plagicolella*, a sample of 273 leaf mines from BULGARIA, 43°12′45.1″ N 27°56′23.4″ E, 24–30 August 2023, leg. S.R. Hill & J.R. Stonis.
(6) *S. colchica* sp. nov., a sample of 279 leaf mines from GEORGIA, Kutaisi, 42°16′32.6″ N 42°42′36.5″ E–42°17′03.1″ N 42°43′42.1″ E, 25 July 2023, leg. S.R. Hill & J.R. Stonis;
(7) *S. colchica* sp. nov., a sample of 60 leaf mines from GEORGIA, Sairme, 41°54′19.2″ N 42°44′56.5″ E, ca 1050 m, 15 July 2023, leg. J.R. Stonis;
(8) *S. colchica* sp. nov., a sample of 333 leaf mines from GEORGIA, Samtredia, 42°09′47.6″ N 42°20′34.2″ E, 24 July 2023, leg. S.R. Hill & J.R. Stonis;
(9) *S. colchica* sp. nov., a sample of 350 leaf mines from GEORGIA, Adjara AR, Kobuleti, 41°49′47.8″ N 41°46′43.9″ E, ca. 10 m, 23 July 2023, leg. S.R. Hill & J.R. Stonis;
(10) *S. colchica* sp. nov., a sample of 141 leaf mines from GEORGIA, Batumi, 41°38′21.9″ N 41°37′44.0″ E–41°42′38.7″ N 41°43′42.1″ E, ca. 25 m, 17–23 July 2023, leg. S.R. Hill & J.R. Stonis;
(11) *S. colchica* sp. nov., a sample of 51 leaf mines from GEORGIA, Adjara AR, SE of Batumi, Mirveti, 41°31′36.4″ N 41°42′58.6″ E, ca. 150 m and Machakhela N.P., Zeda Chkhutuneti, 41°29′05.9″ N 41°51′28.4″ E, ca. 600 m, 18 July 2023, leg. J.R. Stonis;
(12) *S. colchica* sp. nov., a sample of 396 leaf mines from GEORGIA, Adjara AR, Sarpi (type locality of *S. colchica* sp. nov.), 41°31′26.2″ N 41°33′02.1″ E, ca. 80 m, 19–20 July 2023, leg. S.R. Hill & J.R. Stonis;
(13) *S. colchica* sp. nov., a sample of 111 leaf mines from TÜRKIYE, Karadeniz Bölgesi, Artvin Province, Hopa, 41°23′39.4″ N 41°25′02.6″ E, ca. 5 m, and Üçkardeş, 41°29′35.7″ N 41°32′24.2″ E, ca. 70 m, 20 July 2023, leg. J.R. Stonis & S.R. Hill.

In all these localities, all leaf mines found, rather than random examples were taken for statistical analysis including both fresh and old.

Samples from Britain were considered Western, those from Croatia and Bulgaria as Central, and samples from Georgia and northeastern Turkey as Eastern. Sample sizes were 246, 1009, and 1721, respectively. Additionally, to test the Eastern–Western differences and differences between two species, samples from Great Britain, Croatia, and Bulgaria were pooled into the Western pool (*n* = 1255), while the rest of the samples (*n* = 1721) were considered the Eastern pool. All samples from the Western and Central pools belong to *S. plagicolella*, while the samples assigned to the Eastern pool belong to *S. colchica* sp. nov.

Beside these sizeable samples, some other, smaller samples of leaf mines were collected by us from Croatia (Rijeka), Lithuania, Ukraine (Crimea), Georgia (Abkhaz), and Armenia (Sevan) (white dots in Figure 15), but they were not analyzed statistically because of their small size.

Statistical treatment: Egg deposition. No eggs deposited on the upper sides of leaves were detected in the Western and Central samples, while from the Eastern samples, we found that in 183 out of 1721 cases, eggs were deposited on the upper side (10.6%, CI = 9.3–12.2%).

**Figure 16:**
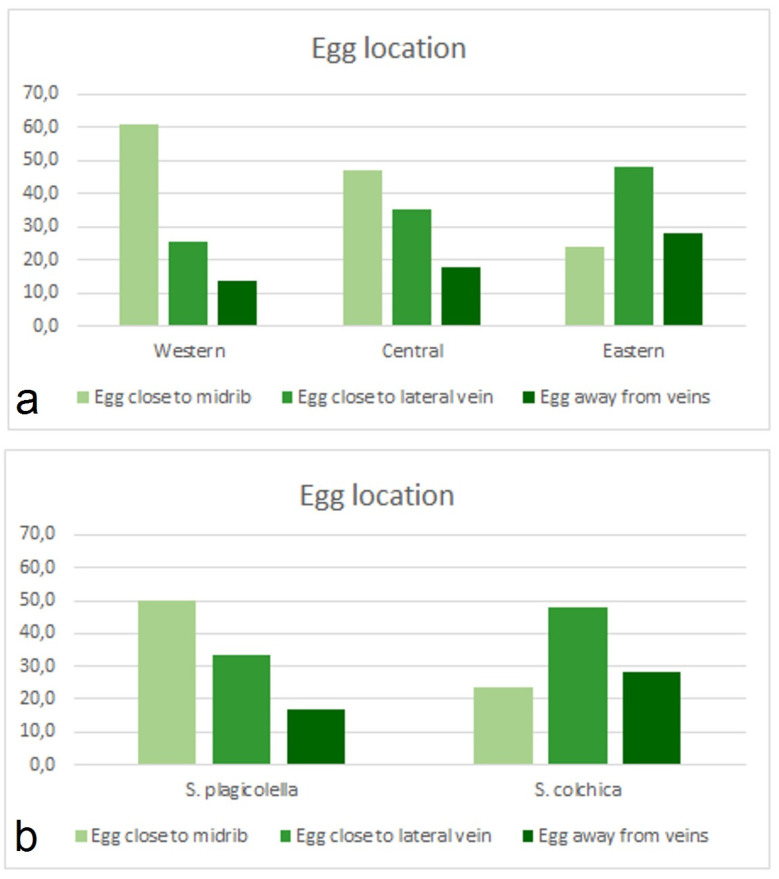
Distribution of observed cases of egg location in relation to leaf veins: (**a**) egg locations in the Western, Central, and Eastern samples; and (**b**) egg locations of the European *Stigmella plagicolella* (the Western and Central samples) and the Caucasian *S. colchica* sp. nov. (the Eastern samples).

Location of eggs in relation to leaf veins (Figure 16). The number of findings with different egg locations in the Western, Central, and Eastern samples was not consistent (χ^2^ = 499.3, df = 4, *p* < 0.0001). A Western-to-Eastern trend was clearly expressed (Figure 16), with a decreasing proportion of eggs located close to the midrib (G = 232.2, *p* < 0.0001), and an increasing proportion of eggs close to the lateral vein (G = 72.7, *p* < 0.0001) and they were unrelated to leaf veins (G = 55.4, *p* < 0.0001) in the Eastern sample. All differences of Stigmella colchica sp. nov. (i.e., Eastern samples) from *S. plagicolella* (the rest of the samples) are significant *p* < 0.0001.

**Figure 17:**
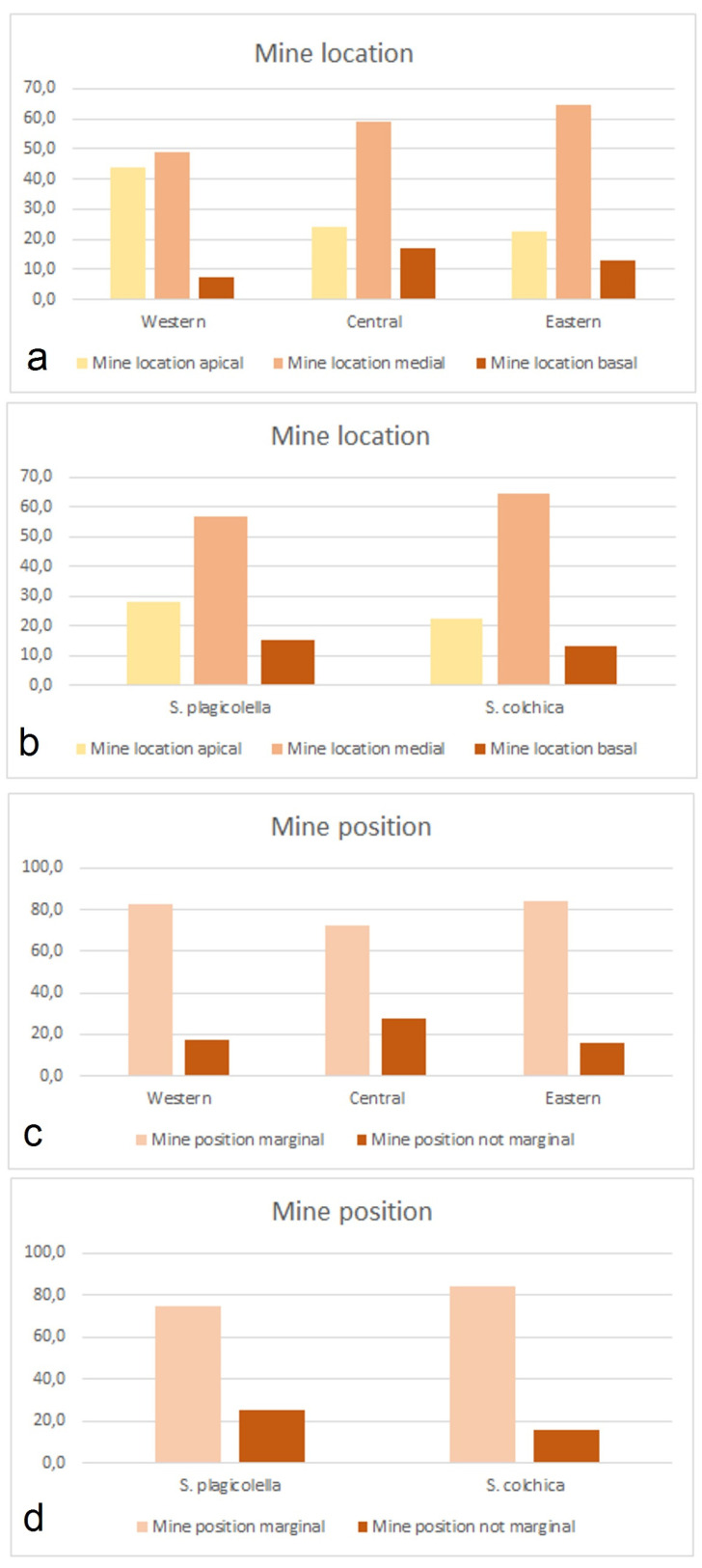
Distribution of observed cases of leaf-mine location in relation to leaf parts: (**a**) mine location in the Western, Central, and Eastern samples; (**b**) mine location in comparison of the European *Stigmella plagicolella* (the Western and Central samples) and the Caucasian *S. colchica* sp. nov. (the Eastern samples); (**c**) mine position in the Western, Central, and Eastern samples; and (**d**) mine position in comparison of the European *Stigmella plagicolella* (the Western and Central samples) and the Caucasian *S. colchica* sp. nov. (the Eastern samples).

Leaf-mine location in respect to leaf parts (apical, medial, and basal) (Figure 17). Compared locations were different (χ^2^ = 263.6, df = 4, *p* < 0.0001) in our geographically distinct samples (Figure 17). The proportion of apical leaf mines in the Western sample (28.0%, CI = 25.6–30.6%) compared to that in the Eastern sample (22.6%, CI = 20.7–24.6%) was significantly higher (G = 45.8, *p* < 0.0001). A similar situation was present with the proportion of basally located mines, which in the Western sample were higher than in the Eastern samples (G = 15.3, *p* < 0.005). In contrast, the proportion of leaf mines deposited in medial parts of the leaves was higher in the Eastern samples (64.5%, CI = 62.2–66.7% versus 57.0%, CI = 54.2–60.0%, G = 21.4, *p* < 0.0001).

When we tried to compare the Western–Eastern gradient with a separate Central sample, the proportion with a basal location was highest in the Central samples: 7.3% (4.9–11.3% in the Western sample), 16.8% (14.7–19.3% in the Central samples), and 12.9% (11.4–14.6% in the Eastern samples); the differences in proportions are significant (G = 17.9, *p* < 0.001).

Mine position in respect to the leaf edge (Figure 17). Compared positions (marginal and not marginal, i.e., not reaching the leaf margin) were also different (χ^2^ = 88.6, df = 2, *p* < 0.0001). The samples of *S. colchica* (i.e., the Eastern samples) were characterized by a higher proportion of marginal leaf mines and a lower proportion of mines located in the leaf interior (G = 42.7, *p* < 0.0001).

**Figure 18:**
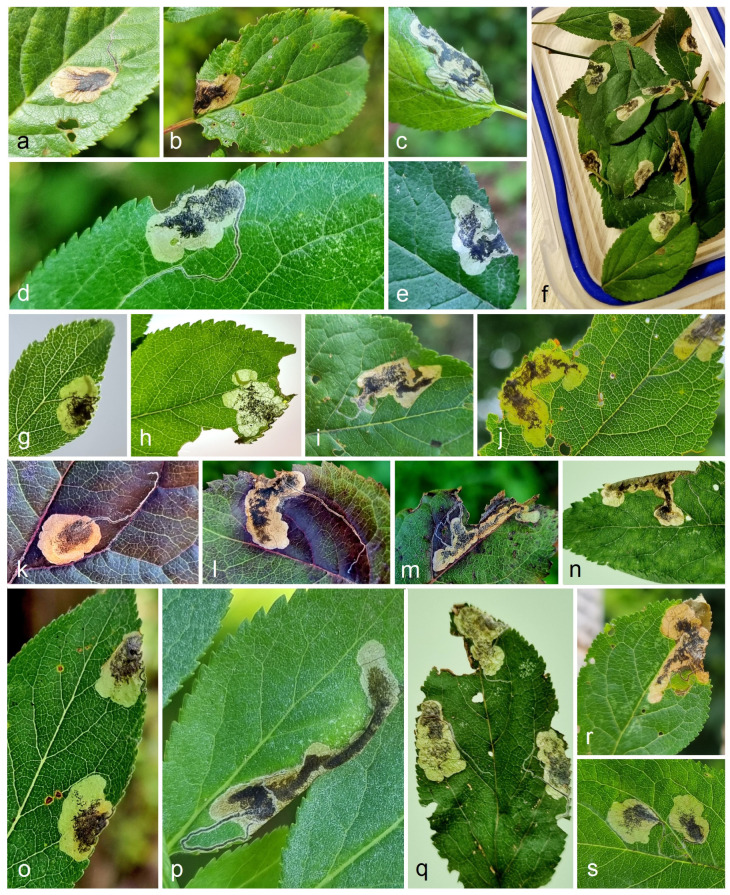
An example of a small fraction of the studied leaf-mine sample of *Stigmella colchica* sp. nov. (**a**–**s**) from the Colchis Lowland in Georgia.

**Figure 19:**
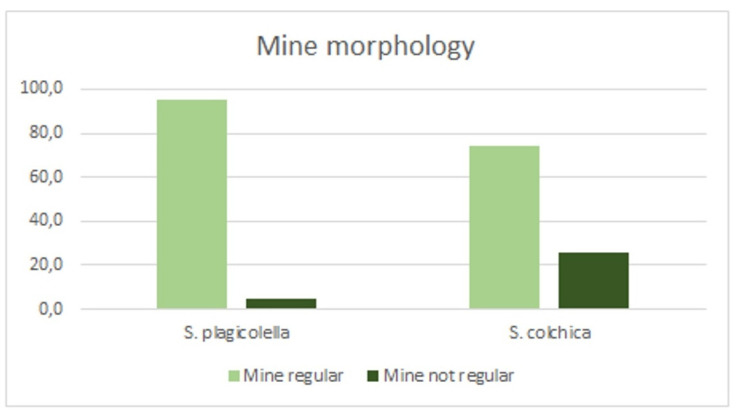
The ratio of regular leaf mines to irregular ones (i.e., those that are clearly irregular and transitional).

Mine morphology: The distribution of leaf mines of a regular shape and that of an irregular shape (i.e., clearly irregular and at least transitional/intermedial leaf mines) differed between the Eastern (*S. colchica* sp. nov.) and Western samples (*S. plagicolella*) (χ^2^ = 292,5, df = 1, *p* < 0.0001). Thus, the proportion of the regular mines in *S. plagicolella* was significantly higher than in *S. colchica* sp. nov. (Figure 18 and Figure 19), being 95.1% (93.7–96.1%) versus 74.0 (71.8–76.0%), with an inverse proportion of mines that were not regular (G = 257.0, *p* < 0.0001).

## 4. Discussion

Although leaf mines of the Caucasian *S. colchica* sp. nov. closely resemble those of the European *S. plagicolella*, making it typically challenging or impossible to distinguish individual leaf mines without locality data, the statistical analysis reveals a discernible trend between the leaf mines of *S. colchica* sp. nov. and *S. plagicolella*. Importantly, our study clearly demonstrated that the Caucasian *S. colchica* sp. nov. and the European *S. plagicolella* can be easily differentiated based on numerous morphological characters of the male genitalia. Moreover, the results of our examination of molecular sequences well supported the morphological data.

According to Hebert et al. [45], mtDNA CO1 is an appropriate tool in species recognition, as more than 98% of species pairs are characterized by greater than 2% sequence divergence. This presumption is used for application by other researchers, e.g., Xue et al. Ref. [46] separated and described a new hesperiid species (Lepidoptera) based on 2.8% pairwise difference of CO1. In our case, the difference between inter- and intraspecific divergences of *S. colchica* sp. nov. and *S. plagicolella* fluctuated between 3.5% and 6.02%. Moreover, the utilized delimitation algorithms reliably clustered them separately (Figure 14), as does the mitotype network (Figure 13). Consequently, we can say with certainty that the molecular characters distinguish *S. colchica* specimens from *S. plagicolella*.

Both analyzed species were characterized by high mitotype diversity, the probability of randomly selecting two different mitotypes from the same population [47] was high (*S. colchica* Hd = 0.85 ± 0.037, *S. plagicolella* Hd = 0.84 ± 0.035). By contrast, nucleotide diversity—the average number of nucleotide differences per site between two randomly chosen DNA sequences [48]—was relatively low (*S. colchica* π = 0.006 ± 0.0004, *S. plagicolella* π = 0.003 ± 0.0003). This was also evident from the mitotype network, which showed mostly single-nucleotide differences between mitotypes within the species (Figure 13). Such a contradiction has been often observed during other mitochondrial DNA studies of insects [32,49,50,51], crustaceans [52], molluscs [53], fish [54,55], mammals [56], etc., and such mitotype and nucleotide diversity combination is characteristic of populations that have expanded over a relatively short period of time from a small effective population size to their currently inhabited areas [57]; this assumption was approved by the Tajima‘s D, Fu and Li‘s D, Fu and Li‘s F neutrality tests in the case of *S. plagicolella*. Unlike some other Lepidoptera (e.g., [58]), we suppose that pygmy leaf-mining moths are weak flyers and do not migrate; therefore, their rapid expansion might be related to the human-assisted transportation of their host plants.

The highest level of genetic diversity was detected in two countries: Georgia (eight mitotypes of *S. colchica* sp. nov.) and Bulgaria (ten mitotypes of *S. plagicolella*). This is likely due to the large number of samples collected and analyzed from these countries, leading to the conclusion that more careful studies in other places of the world would also increase the genetic diversity of the species studied there.

The discovery of new species such as *S. colchica* sp. nov. in the Caucasus is significant for various reasons, particularly due to its potential pest status affecting both wild and cultivated plum trees. *S. colchica* sp. nov. falls into the category of potential pests that can cause damage to plum trees, particularly in cases of severe infestations. Beyond the impact on cultivated host plants, the identification of this new species in the Caucasus enhances our understanding of the region, contributing to its historical biogeography and providing insights into the evolutionary processes that shaped the Caucasian and European biota. While the discovery of *S. colchica* sp. nov. may offer valuable insights into evolutionary processes, we currently refrain from speculating on the origin of the seemingly allopatric species, *S. colchica* sp. nov. and *S. plagicolella*.

Ongoing monitoring is necessary to understand *S. colchica*’s behavior, preferred host plants, and potential threats to cultivated plums. The close geographical proximity of the Caucasus to Europe underscores the importance of continuous monitoring of this newly identified pest in the Caucasus region, particularly considering the potential influence of climate change on pest distribution and the imperative to prevent unintentional pest spread.

## Data Availability

The molecular data presented in this study can be found in online repositories (Genbank and BOLD). Open access depositories of the collection material (physical specimens and their genitalia mounts, including of leaf-mine samples of *Stigmella colchica* sp. nov. and *S. plagicolella*) are listed in the article.

## Supplementary Materials

The following supporting information can be downloaded at: https://www.mdpi.com/article/10.3390/insects15030198/s1, Table S1: The CO1-5′ mitotypes of *Stigmella colchica* sp. nov. (SC1–SC8) and *S. plagicolella* (SP1–SP20), sequences and GenBank accession IDs, countries of origin (Sequences obtained during this study are marked with *).
