# Supplementary material for: Revealing a Novel Potential Pest of Plum Trees in the Caucasus: A Species Resembling the European Leaf-Mining Stigmella plagicolella, Nepticulidae
Source: Insects. 2024 Mar 15;15(3):198. doi: 10.3390/insects15030198 (PMC10971272; doi:10.3390/insects15030198)
Supplement: Supplementary file 1 [file insects-15-00198-s001.zip › insects-2904333-supplementary.pdf]

Table 1. The CO1-5′ mitotypes of Stigmella colchica sp. nov. (SC1–SC8) and S. plagicolella (SP1–SP20), sequences and GenBank accession IDs, countries of origin. Sequences obtained during this study are marked with *.

Mitotype	Sequence ID	GenBank accession ID	Country
SC1	SC5540*	PP318246	Georgia
SC2	SC5533*	PP318247	Georgia
	SC5534*	PP318248	
	SC5535*	PP318249	
	SC5537*	PP318250	
	SC5545*	PP318251	
	SC5550*	PP318252	Türkiye
	SC5551*	PP318253	
	SC5552*	PP318254	
	SC5553*	PP318255	
SC3	SC5522*	PP318230	Georgia
	SC5523*	PP318231	
	SC5541*	PP318232	
	SC5547*	PP318233	
SC4	SC5544*	PP318234	Georgia
	SC5548*	PP318235	
SC5	SC5526*	PP318226	Georgia
	SC5527*	PP318227	
	SC5528*	PP318228	
	SC5529*	PP318229	
SC6	SC5531*	PP318236	Georgia
	SC5532*	PP318237	
	SC5536*	PP318238	
SC7	SC5538*	PP318240	Georgia
	SC5539*	PP318241	
	SC5542*	PP318242	
	SC5543*	PP318243	
	SC5546*	PP318244	
	SC5549*	PP318245	
SC8	SC5530*	PP318239	Georgia
SP1	GMBUA513-14	-	Bulgaria
	GMBUB1564-14	-	
	GMBUC1528-14	-	
	GMBUC1542-14	-	
	GMBUC1548-14	-	
	GMBUC1830-14	-	
	GMBUF1923-14	-	
	GMBUF1925-14	-	
	GMBUF1926-14	-	
	GMBUF1928-14	-	
	GMBUF1930-14	-	
	GMBUF1933-14	-	
	GMBUF1937-14	-	
	GMBUF1939-14	-	

1

	GMBUF1940-14	-	
	GMBUF1943-14	-	
	GMBUG1264-14	-	
	GMBUG2159-14	-	
	LEEUA014-11	JN283998	Denmark
	SP5560*	PP318262	Croatia
	SP5561*	PP318263	
	SP5570*	PP318264	Lithuania
SP2	GMBUF1938-14	-	Bulgaria
SP3	GMGMP5652-18	-	Germany
	NEPSE004-11	KX281339	Belgium
SP4	GMBUC1829-14	-	Bulgaria
SP5	GMBUF1918-14	-	Bulgaria
SP6	GMBUC1529-14	-	Bulgaria
SP7	SP5562*	PP318265	Croatia
SP8	GMBUB1792-14	-	Bulgaria
	GMBUC1550-14	-	
	GMBUC1827-14	-	
	GMBUC1828-14	-	
	GMBUF1931-14	-	
	GMBUF1934-14	-	
	GMBUF1941-14	-	
	GMBUF1942-14	-	
	GMBUG2162-14	-	
SP9	GMBUF1924-14	-	Bulgaria
SP10	GMBUD1521-14	-	Bulgaria
SP11	GMBUG1269-14	-	Bulgaria
SP12	LEFII214-11	KT782519	Finland
	LEFIJ1040-11	KX049419	
	LEFIJ1413-12	KX049533	
SP13	GMBUC1558-14	-	Bulgaria
SP14	NEPSE019-11	-	France
SP15	PHLAJ204-14	-	Austria
SP16	GMGMJ1478-14	-	Germany
	GMGMN826-14	-	
	NEPSE018-11	-	France
	NLLEA1517-20	-	The Netherlands
	SP5554*	PP318257	Great Britain
	SP5555*	PP318256	
	SP5556*	PP318258	
	SP5557*	PP318259	
	SP5558*	PP318260	
	SP5559*	PP318261	
SP17	NEPTA1259-15	-	Italy
	NEPTA630-13	-	France
	NEPTA631-13	-	
SP18	NEPSE003-11	-	Belgium
SP19	NEPTA632-13	-	Greece
SP20	NEPTA633-13	-	The Netherlands
